# Toward an economy of wellbeing: The economic impact of the Welsh healthcare sector

**DOI:** 10.3389/fpubh.2022.953752

**Published:** 2022-10-26

**Authors:** Timotej Jagrič, Christine Brown, Dušan Fister, Oliver Darlington, Kathryn Ashton, Mariana Dyakova, Mark A. Bellis, Vita Jagrič

**Affiliations:** ^1^Institute of Finance and Artificial Intelligence, Faculty of Economics and Business, University of Maribor, Maribor, Slovenia; ^2^European Office for Investment and Health and Development, World Health Organization, Venice, Italy; ^3^Public Health Wales, WHO Collaborating Centre on Investment for Health and Well-being, Capital Quarter 2, Cardiff, United Kingdom

**Keywords:** input-output analysis, healthcare sector, Wales, impact analysis, economy of wellbeing

## Abstract

Population health and wellbeing is both a result, as well as a driver, of economic development and prosperity on global, European, national and sub-national (local) levels. Wales, one of the four United Kingdom (UK) nations, has shown a long-term commitment to sustainable development and achieving prosperity for all, providing a good example of both national and sub-national level, which can be useful for other European countries and regions. In this paper, the economic importance of the healthcare sector to the Welsh economy is explored. We use a large number of data sources for the UK and Welsh economy to derive an economic model for 2017. We estimate output, income, employment, value-added, and import multipliers of the healthcare sector. Results suggest that the healthcare sector has an above average contribution in four explored economic aspects of the Welsh economy (output, income, employment, value-added), according to its impact on the surrounding economic ecosystem. Also, it is below average regarding leaking through imports. The multipliers' values offer empirical evidence when deciding on alternative policy actions. Such actions can be used as a stimulus for encouraging regional development and post-COVID economic recovery. Our study refers to the Welsh healthcare sector's economic impact as a whole. Therefore, we suggest investigating the economic impact of individual healthcare providers in the future.

## Introduction

The uncertain and dynamic time we live in, facing challenges of epidemics, climate change, economic instability, societal disruption and escalating inequalities, requires an urgent and explicit recognition of the value and wider economic benefits of protecting, improving and caring for the health and wellbeing of people and communities. Population health is both a result of, as well as a driver of, economic development and prosperity on a global, European, national and sub-national (local) level.

Wales, one of the four United Kingdom (UK) nations, has shown a long-term commitment to sustainable development and achieving prosperity for all through relevant legislation (Well-being of Future Generations Act - WFGA^1^, Socio-economic Duty - SED^2^) and policy levers (Programme for Gov^3^, A Healthier Wales^4^) to ensure the health and wellbeing of current and future generations. It provides a good example of both national (devolved nation with autonomous health and social care sector) and sub-national level (as part of the wider UK system), which can be useful for other European countries and regions. Wales has an explicit commitment to improving population health through its health related legislation, policies and programmes of work. However, the Welsh National Health Service (NHS) is still struggling to cope with demand and the needs of the population, with health inequalities and waiting lists exacerbated by the COVID-19 pandemic, as well as the direct and indirect implications of climate change, rising cost of living and the UK departure from the European Union (Brexit) [see e.g., (1)].

The economic ecosystem is an interconnected supply and demand system of different industries. One of these is the healthcare sector. Due to its economic linkages to other sectors, changes in the demand for its services cause a knock-on effect on the whole economic ecosystem having direct, indirect, and induced effects. One way of monitoring the impact is by measuring the change in the economy's output, value-added, income, employment, and import as input-output (IO) multipliers. The impact and economic importance of the healthcare sector in the economic ecosystem are being broadly studied in the literature from different perspectives, naming some (2–10).

From the economic point of view, the COVID-19 pandemic is a health-triggered economic crisis. Its economic consequences are immense and have immediately called for policy actions (11–14). Health policy is primarily devoted to achieving public health goals. However, we argue that it is also justified to consider the economic effects of public health expenditures and the healthcare sector's activity when designing policy actions. These actions can simultaneously support multiple societal priorities, one of them being the economic recovery in the post-COVID-19 period.

Further, health policy actions can support policies from other fields. Due to trends like deindustrialization, the weakening of social infrastructure, loss of employment opportunities, or population decrease, growing disparities between the capitals and the rural regions can be observed widely in Europe and elsewhere (15). Addressing development disparities with new job opportunities and more robust healthcare infrastructure can also result from health policy action. Not to forget that providing healthcare services results in first place in non-economic effects, namely, improved population health outcomes. Illness costs suppress the economic ecosystem, thus improved health is favorable from the economic perspective (16–20) as it results in a healthier and more productive workforce, less absenteeism and less lost working days due to illness.

In developed economies, the size of the healthcare sector is generally large, however its economic importance differs by country due to the particular economic interrelation to the local economy. Further, due to structural changes, it also varies over time. Therefore, empirical evidence for each individual country is needed to estimate the extent of the economic footprint of the healthcare sector. In this paper we have chosen to study an example of a small, high-income country, namely, Wales. This case study aims to quantify the economic importance of the Welsh healthcare sector to the Welsh economy. The studies' results bring novelty to the international literature body. To evaluate the obtained empirical results and its robistness, we are benchmarking them to the previous empirical studies from other countries (8, 10, 21) and most importantly, compare them to the forecasted value of econometric models developed in an international study (21).

The rest of the analysis is structured as follows. First, data is described and where it was gathered from. Next, methodological note is provided. After it, results are presented and interpreted. Finally, we give our conclusions and discuss the policy implications.

## Materials and methods

### Data on the Welsh and the UK economy

In this research, we used input-output (IO) tables. The economic ecosystem consists of a group of industries, which both produce goods and consume goods produced by other industries. IO analysis thus reflects the flow of products from producers to consumers, where each industry is considered in both positions starting from an interindustry transactions table (22). For our research an IO table was needed at the Welsh level. The most recent IO table for Wales was published in 2007 (23). In this analysis, multiple data sources presented in Table 1 were used to create a more recent IO table. We used the 2017 United Kingdom IO analytical table as a general benchmark, alongside the 2007 Welsh IO table. Further, additional data variables from different sources were used for the database creation, as described in Table 1.

**Table 1:** Data description and sources.

**Data**	**Year**	**No. of sectors**	**Source**	**Classification, and details**
UK IO analytical table	2017	105	ONS^a^	CPA, Domestic use, basic prices, product-by-product, £ million
Welsh IO table	2007	88	(23)^b^	SIC2003, Domestic use, basic prices, £ million
Employment by industry (UK, Wales)	2017	88	Nomis^c^	SIC2007, No. of employees Business Register and Employment Survey
Final consumption expenditure by government (UK, Wales)	2017–2018	10 (16)	ONS^d^	COFOG, £ million, EU transactions recorded on net basis, as they include: (1) GNI and VAT-based EU contributions, (2) EU foreign aid contributions, (3) EU receipts from Common Agricultural Policy and Structural Funds.
National household final consumption expenditure NUTS1 (UK, Wales)	2017	12 (42)	ONS^e^	COICOP commodities, UKL NUTS code, £ million, in current market prices, experimental statistics.
Regional Gross fixed capital formation, NUTS1	2017	10 (11)	ONS^f^	SIC2007, UKL NUTS code, £ million. Production sector (sectors BCDE) further divided into manufacturing (C).
Exports of goods (Regional trade statistics)	2017	10	HMRC^g^	SITC, £ million
Total value of service exports from the UK by NUTS1 area, industry, and destination	2017	13	ONS^h^	SIC2007, £ million, UK Balance of Payments - The Pink Book
Taxes less subsidies on products	2017	Total	ONS^i^	TLL ITL code, £ million
Compensation of employees by industry	2017	31	ONS^j^	SIC2007, TLL ITL code, £ million Estimates of workplace based GVA.
Gross operating surplus and mixed income	2017	31	ONS^k^	SIC2007, TLL ITL code, £ million
Taxes less subsidies on production by industry at current basic prices	2017	31	ONS^l^	SIC2007, TLL ITL code, £ million Estimates of workplace based GVA.
Regional gross value added (ITL1 current price estimates)	2017	81	ONS^m^	TLL ITL code, £ million
Regional gross value added in Wales by industry	2017	81	StatsWales^n^	SIC2007, £ million, current prices.
Approximate gross value added at basic prices (aGVA)	2017	73	ONS^o^	SIC2007, £ million, Data source from Annual Business Survey (ABS).
Total output (total turnover) at basic prices (Non-financial business economy, UK regional results: Sections A to S)	2017	19 (77)	ONS^p^	SIC2007, £ million, Data source from Annual Business Survey (ABS).
UK and Wales Gross domestic product (GDP) at current market prices	2017	Total	ONS^q^	TLL ITL code, £ million
UK and Wales Gross domestic product (GDP) per head at current market prices	2017	Total	ONS^r^	TLL ITL code, £ million
UK and Wales Total GDHI at current basic prices	2017	Total	ONS^s^	UKL NUTS code, £ million
ITL1 implied deflators	2017	81	ONS^t^	SIC2007, TLL ITL code

a https://www.ons.gov.uk/economy/nationalaccounts/supplyandusetables/datasets/ukinputoutputanalyticaltablesdetailed.

b https://www.cardiff.ac.uk/_data/assets/pdf_file/0010/698869/input-output-tables-2007-final-30-6.pdf.

c https://www.nomisweb.co.uk/query/construct/components/stdListComponent.asp?menuopt=12&subcomp=100.

d https://www.ons.gov.uk/economy/governmentpublicsectorandtaxes/publicsectorfinance/datasets/countryandregionalpublicsectorfinancesexpendituretables.

e https://www.ons.gov.uk/economy/regionalaccounts/grossdisposablehouseholdincome/datasets/regionalhouseholdfinalconsumptionexpenditure.

f https://www.ons.gov.uk/economy/regionalaccounts/grossdisposablehouseholdincome/adhocs/10949regionalgrossfixedcapitalformationnuts1andnuts22000to2018.

g https://www.uktradeinfo.com/trade-data/regional/2020/uk-regional-trade-in-goods-statistics-fourth-quarter-2020/.

h https://www.ons.gov.uk/businessindustryandtrade/internationaltrade/datasets/internationalexportsofservicesfromsubnationalareasoftheuk.

i https://www.ons.gov.uk/economy/grossdomesticproductgdp/datasets/regionalgrossdomesticproductallnutslevelregions.

j https://www.ons.gov.uk/economy/grossvalueaddedgva/datasets/nominalregionalgrossvalueaddedbalancedperheadandincomecomponents.

k https://www.ons.gov.uk/economy/grossvalueaddedgva/datasets/nominalregionalgrossvalueaddedbalancedperheadandincomecomponents.

l https://www.ons.gov.uk/economy/grossvalueaddedgva/datasets/nominalregionalgrossvalueaddedbalancedperheadandincomecomponents.

m https://www.ons.gov.uk/economy/grossvalueaddedgva/datasets/nominalandrealregionalgrossvalueaddedbalancedbyindustry.

n https://statswales.gov.wales/Catalogue/Business-Economy-and-Labour-Market/Regional-Accounts/Gross-Value-Added-GDP/gvainwales-by-industry.

o https://www.ons.gov.uk/businessindustryandtrade/business/businessservices/datasets/uknonfinancialbusinesseconomyannualbusinesssurveyregionalresultssectionsas.

p https://www.ons.gov.uk/businessindustryandtrade/business/businessservices/datasets/uknonfinancialbusinesseconomyannualbusinesssurveyregionalresultssectionsas.

q https://www.ons.gov.uk/economy/grossdomesticproductgdp/datasets/regionalgrossdomesticproductallnutslevelregions.

r https://www.ons.gov.uk/economy/grossdomesticproductgdp/datasets/regionalgrossdomesticproductallnutslevelregions.

s https://www.ons.gov.uk/economy/regionalaccounts/grossdisposablehouseholdincome/datasets/regionalgrossdisposablehouseholdincomegdhi.

t https://www.ons.gov.uk/economy/grossvalueaddedgva/datasets/nominalandrealregionalgrossvalueaddedbalancedbyindustry.

Two UK IO tables are published by Office for National Statistics (ONS), the (1) product by industry, and the (2) product by product IO table (the latter was considered in this study). The product by product table follows the statistical Classification of Products by Activity (CPA). Monetary terms are specified in the current basic prices (£ million); the intermediate demand is symbolized by a symmetric 105x105 matrix and two additional matrices, i.e., the final demand (FD) and final payments (FP) are attached on the right and lower borders (elements of these are outlined in Table 2).

**Table 2:** Final demand matrix for both UK and Wales IO tables.

**Final demand matrix**		**Final payments matrix**	
**IO table UK**	**IO table Wales**	**IO table UK**	**IO table Wales**
Final consumption expenditure	–	Use of imported products, cif	Imports RUK
Final consumption expenditure by government	Government	–	Imports ROW
Final consumption expenditure by households	Consumers	Compensation of employees	Disposable Income (Employees & Self emp)
Final consumption expenditure by NPISH	NPISH	Gross operating surplus and mixed income	Gross Operating Surplus (excluding mixed income)
–	Daytrippers	–	Income & Self employment Tax & NIC
–	Stock200	Taxes less subsidies on production	Taxes less subsidies on production
Gross fixed capital formation	GFCF	Taxes less subsidies on products	Taxes on Products
–	Tour 1–3	Gross value added	–
–	Tour 4+		
–	Tour Intl		
–	Tour Bus		
Changes in inventories	–		
Acquisitions less disposals of valuables	–		
Export of goods to EU	–		
Exports of goods to rest of the world	Exports ROW		
Exports of services	–		
–	Exports RUK		

Sources: ONS and (23). RUK, Rest of UK; ROW, Rest of World.

The 2007 Welsh IO table differs slightly from the UK's, as it incorporates 88 sectors, as outlined in the report by Jones et al. (23). FD and FP differences between the UK and Welsh IO table are denoted in Table 2. For regionalization of an IO table, standardized employment data are required on both levels (24). Therefore, in the Welsh case, data is needed, on the UK and Weles level. Standardization is crucial for the test of the tables as well, these were, prior to use, first adjusted at our best efforts among different standards, such as Standard Industrial Classification (SIC 2003 and SIC 2007), Classification of the Functions of Government (COFOG), Classification of Individual Consumption by Purpose (COICOP), Standard international trade classification (SITC).

The element of final consumption expenditure was further divided by (1) government, by (2) households, and by (3) non-profit organizations serving households (NPISHs). In the Welsh case, the third element was missing. Final consumption by government was ranked into three different categories: (1) total, (2) current and (3) capital expenditure. There were 10 individual industry sectors, some of which were further divided into sub-sectors. Altogether, data for 16 industry sectors on government consumption were available. Data on final consumption expenditure by households was also available at the exceptional sectoral division. Although the statistics were experimental, ONS have published the regional household final consumption expenditure by COICOP commodities. There were 12 large groups of products and further 42 sub-groups of products outlined by the COICOP commodities division. Finally, consumption for 20 basic commodities was outlined as well.

Regional Gross Fixed Capital Formation (GFCF) is an aggregate measure for Wales, as there are only 11 SIC2007 industry groups available. Consequently, aggregation is applied, i.e., health sector is aggregated with public administration and education sectors; export/import of goods are no different. The HMRC web portal for regional trade statistics publishes data for 10 different SITC sections, total export of services is available for 13 highly aggregated groups. Unfortunately, no interregional trade by industry data was found that could act as a superior information.

Regional taxes less subsidies on products for Wales is available at total aggregate level (a single value); these are calculated as the sum of VAT and other taxes on products, subtracted by subsidies on products. Taxes less subsidies in production are calculated similarly, but are readily available at much more detailed level, i.e., 31 industry sectors. After all, these carry an important information value, by representing the difference between Welsh Gross Value Added (GVA) (B) and Welsh GDP. Gross operating surplus and mixed income are calculated as a sum of seven elements.

International Territorial Levels—Level 1 (ITL1) current price estimates by ONS and regional GVA in Wales by industry by StatsWales are identical figures, but differentiate from approximate gross value added (aGVA) obtained from ONS. For the majority of sectors aGVA deviates from GVA. The total output at basic prices (which is equal to total use, i.e., the first is sum of rows and the second sum of columns) is found to be available on the level of 19 SIC2007 industry groups, which can be parceled into 77 industry sectors. The regional data by Annual Business Survey (ABS) covers the non-financial industry sectors only. As well, some other industries, such as mining, electricity, public administration and defense, and education are omitted or given an information on higher aggregated group.

Regional and national GDPs are calculated as GVA + VAT on products + other taxes on products - subsidies on products; also, GDP per head is calculated to compare economies between the regions and a nation themselves; here, implied sectoral deflators provide a fair comparison between 2007 and 2017 prices.

### Generation of Welsh IO table and multipliers calculation

For this study, the greatest methodological challenge was to derive a Welsh IO table. Some of the standard procedure's steps from the literature had to be adjusted to the Welsh individual case. The IO table was generated using the mechanical manipulation and insertion of superior data. We employed a modified GRIT methodology to derive Welsh IO table from the single UK IO table by implementing the following steps:

- adjustment to the UK table,
- adjustment to the Welsh import data,
- definition of Welsh sectors,
- definition of prototype transactions tables,
- GRAS optimization,
- definition of final transactions tables.

In the case of Queensland in 1979, a process regionalizing a national IO table, later known as Generation of Regional Input-Output Tables (GRIT method) was developed (24). It has been applied for many cases, for example Greece (25), Germany and the Czech Republic (26), and the Mediterranean region (27). Additionally, the purpose of GRIT regionalization has been verified using the Monte Carlo simulation (28). Although the classic GRIT methodology is a 15-step process (24), the methodology has been often tailored to the needs of researchers.

Mattas et al. (25, 29) outlined the complete (customized) methodology of GRIT regionalization along with the programming code. GRIT starts from the national IO table **T**. The national table **T** has three elements: (1) the central interindustry matrix **Z** (*n x n*), (2) the matrix of final demand **fd** (*n x h*), and (3) the vector of final payments **fp** (*l x n*). The sum of interindustry matrix and final demand **fd** can be denoted as follows (29):


$$\begin{array}{l} \text{x}=\text{Z}\cdot \text{i}+\text{f}\text{d}, \end{array}$$

where the sum **x** is of dimension (1 *x n*) and **i** is a ones vector. The sum **x** is identical to the sum of interindustry matrix and final payments (except transposed), as follows:


$$x^{\prime }=i^{\text{T}}\cdot Z+fp.$$

Hence, the IO table is balanced. The step 1 of the GRIT procedure is to ensure that the national technical coefficients are calculated as follows:


$$\begin{array}{l} {\text{A}}_{nxn}^{\left(0\right)}=\text{Z}\cdot {\hat{\text{x}}}^{-1}, \end{array}$$

where the ${\text{A}}_{nxn}^{\left(0\right)}$ depicts the initial datum technical coefficients. Similarly, the initial datum matrix of primary inputs is denoted as ${\text{f}\text{p}}_{lxn}^{\left(0\right)}$, withholding the *l*-elements of the primary inputs. Since Jensen et al. (24) denote the original matrix of primary inputs as ${\text{B}}_{lxn}^{\left(0\right)}$ and this causes confusion with Leontief inverse matrix, we renamed it to ${\text{f}\text{p}}_{lxn}^{\left(0\right)}$. Additionally, as suggested by Mattas et al. (29), the calculation of technical coefficients is postponed from the step 1 to the step 4. Hence, step 2 and 3 were implemented on the original (interindustry) matrix.

Step 2 is devoted to the prices adjustments (24). For constant prices this process is conducted using the sectoral implied deflators. We have not employed any specific corrections to the obtained national IO table. Step 3 is organized to first identify and outline the primary (P), secondary (S) and tertiary (T) industry sectors, and hence primary, secondary and tertiary sub-matrices of national technical coefficients matrix ${\text{A}}_{nxn}^{\left(0\right)}$. Next, partitioning of the so-called competitive and non-competitive imports within the secondary sub-matrix ${\text{S}}_{sxn}^{\left(0\right)}$ follows, where the *s* denotes number of secondary industry sectors. Finally, a row vector of imports denoted as **m**_1*xn*_, i.e., an extraction of the matrix of primary inputs ${\text{f}\text{p}}_{lxn}^{\left(0\right)}$, is allocated and next distributed over the secondary sectors as follows:


$$d_{1}=i^{\text{T}}\cdot S^{\left(0\right)},$$

where the identity vector is of dimension **i**^T^(1 *x s*) and the **d**_1_ (1 *x n*) represents the sum of column elements of the secondary sub-matrix **S**^(0)^. On the other hand, Mattas et al. (29) treat the **m**_1*xn*_ to include only the secondary sectors and thus designate it as $m_{1xn}^{\left(S\right)}$. We employed the original scenario by Jensen et al. (24) and defined the sum of secondary sub-matrix and imports vector as follows:


$$\begin{array}{l} {\text{d}}_{2}={\text{d}}_{1}+\text{m}, \end{array}$$

where **d**_2_ is again of dimension (1 *x n*). The two sums, i.e., **d**_1_ and **d**_2_ are used to formalize the corrected secondary sub-matrix **S**^(1)^ as follows:


$$\begin{array}{l} {\text{S}}^{\left(1\right)}={\hat{\text{d}}}_{2}\cdot {\hat{\text{d}}}_{1}^{-1}\cdot {\text{S}}^{\left(0\right)}, \end{array}$$

where the superscript denotes diagonalized matrices of dimensions (*n x n*). By replacing the original sub-matrix **S**^(0)^ with the corrected sub-matrix **S**^(1)^, one obtains:


$$\begin{array}{l} {\text{A}}_{nxn}^{\left(1\right)}. \end{array}$$

In our case, instead of Jensen's original method, we employed a similar scenario described by Mattas et al. (29), as follows:


$$\begin{array}{l} {\text{S}}^{\left(1\right)}={\text{S}}^{\left(0\right)}\cdot {\hat{\text{d}}}_{2}\cdot {\hat{\text{d}}}_{1}^{-1}. \end{array}$$

One must note that we have not been operating with the national technical coefficients matrix (relative numbers) ${\text{A}}_{nxn}^{\left(0\right)}$ and ${\text{A}}_{nxn}^{\left(1\right)}$, but instead with the national I-O table (nominal numbers) ${\text{Z}}_{nxn}^{\left(0\right)}$ and ${\text{Z}}_{nxn}^{\left(1\right)}$. Hence, the update of ${\text{A}}_{nxn}^{\left(1\right)}$ with corrected sub-matrix **S**^(1)^ is meant to be ${\text{Z}}_{nxn}^{\left(1\right)}$. Simultaneously, the primary inputs ${\text{f}\text{p}}_{lxn}^{\left(0\right)}$ were updated to the ${\text{f}\text{p}}_{lxn}^{\left(1\right)}$.

Step 4 are adjustments for non-competitive regional imports. Sectors that are non-existent in the region, i.e., sectors with zero regional employment, are zeroized; consequently, a transformation of ${\text{A}}_{nxn}^{\left(1\right)}\to \;{\text{A}}_{nxn}^{\left(2\right)}$ is carried out (for our case ${\text{Z}}_{nxn}^{\left(1\right)}\to \;{\text{Z}}_{nxn}^{\left(2\right)}$). Simultaneously, intermediate input coefficients of such sectors are summed column-wise and added to the import row of ${\text{f}\text{p}}_{lxn}^{\left(1\right)}$, thus becoming ${\text{f}\text{p}}_{lxn}^{\left(2\right)}$. No such sectors were found to be existing in our case. As suggested by Mattas et al. (29), we have zeroized the main diagonal to avoid the existence of intra-sectoral flows, i.e., interregional trade:


$$Z^{*}=Z-Z\cdot \hat{i},$$

where the Z^*^ represents the national interindustry matrix with the diagonal deleted. Additionally, we have calculated the national technical coefficients matrix as follows:


$$A=Z^{*}\cdot {\hat{x}}^{-1}.$$

Step 5 is the actual regionalization procedure, in technical terms called scaling. Scaling is performed using the so-called location quotients (24). Authors denote the location quotients with a common symbol **q**_*nxn*_, where the **q**_*nxn*_ can be calculated alternatively in any means, also by diagonalizing the vector **q**_(1*xn*)_. This is then used to update the technical coefficients as follows:


$$\begin{array}{l} {\text{A}}^{\left(3\right)}=\text{q}\cdot {\text{A}}^{\left(2\right)}, \end{array}$$

where the dimension of **A**^(3)^ remains the (*n x n*). We employed the cross-industry location quotients (CILQ), as follows:


$$\begin{array}{l} CILQ_{ij}=\;\frac{E_{i}^{\left(r\right)}/E_{j}^{\left(r\right)}}{E_{i}^{\left(n\right)}/E_{j}^{\left(n\right)}}, \end{array}$$

Where $E_{i}^{\left(r\right)}$ represents the regional employment and $E_{j}^{\left(n\right)}$ the national. The two data on employment are cross-given iteratively to ensure well-diversified location quotients and thus a solid picture of regional economy. Suggested by Mattas et al. (29), a correction by Flegg and Webber (30) was carried as follows:


$$\begin{array}{l} \lambda =\log_{2}{\left(1+\frac{\sum_{i}E_{i}^{\left(r\right)}}{\sum_{i}E_{i}^{\left(n\right)}}\right)}^{\delta }, \end{array}$$

where 0 ≤ δ ≤ 1 is a weighing parameter, outstanding the relative size of the region, to derive the FLQ location quotients:


$$\begin{array}{l} FLQ_{ij}=CILQ_{ij}\cdot \lambda . \end{array}$$

Finally, the **q** = *FLQ*_*ij*_ coefficients are constrained in the range *FLQ*_*ij*_ ≤ 1 and the regional technical coefficients calculated, as suggested **A**^(3)^ = **q**·**A**^(2 )^.

Next the difference between the **A**^(2)^ and **A**^(3)^ should be calculated and summed column-wise:


$$\begin{array}{l} {\text{i}}^{T}\cdot \left[{\text{A}}^{\left(2\right)}-{\text{A}}^{\left(3\right)}\right], \end{array}$$

to update the ${\text{f}\text{p}}_{lxn}^{\left(3\right)}$ by adding the so-calculated sum difference to the import row. Due to the availability of superior data, this and further steps by Jensen et al. (24) were omitted.

By Jensen et al. (24) step 10 is devoted to the manual balancing of the produced regional I-O table, commonly using the RAS updating and balancing technique of I-O tables by Stone (31). RAS is an iterative approach that scales (or re-scales) the existing matrices in order to best comply the table coefficients with column and row sums (constraints or border conditions). A more general procedure called a Generalized RAS (GRAS) is popular. It was applied by Junius and Oosterhaven (32), Temursho et al. (33), and Temurshoev et al. (34). GRAS has a common benefit according to the RAS, i.e., that it can handle non-negative matrices. In general, it is formalized as follows:


$$\begin{array}{l} \text{X}=\hat{\text{r}}\cdot \text{A}\cdot \hat{\text{s}}, \end{array}$$

hence the name RAS ($\hat{\text{r}}\cdot \text{A}\cdot \hat{\text{s}}$). Matrices $\hat{\text{r}}$ and $\hat{\text{s}}$ are correction factors, are diagonalized and contain non-negative numbers on diagonal, while $\text{A}={\text{A}}_{pxp}^{\left(6\right)}$. The first correction factor ($\hat{\text{r}}$) is a row correction factor, while the second ($\hat{\text{s}}$) the column correction factor. Initially, the GRAS method separates the non-negative and negative elements of the matrix **A** into two matrices: **P** and **N**, where the first contains non-negative elements and the second negative values. Further, the absolute value of the second matrix is calculated to update as follows **N** = |**N**|. It holds that **A** = **P − N**. Sequentially, we can formulate:


$$\begin{array}{l} \left(\hat{r}\cdot P\cdot \hat{s}-{\hat{r}}^{-1}\cdot N\cdot {\hat{s}}^{-1}\right)\cdot i=u^{*},\\ i\cdot \left(\hat{r}\cdot P\cdot \hat{s}-{\hat{r}}^{-1}\cdot N\cdot {\hat{s}}^{-1}\right)=v^{*},\; \end{array}$$

where u^*^ and v^*^ follow the next representation and **i** is an identity vector:


$$\begin{array}{l} u^{*}=e\cdot u,\;\\ v^{*}=e\cdot v.\; \end{array}$$

Here, the *e* is derived from the optimal solution definition **Z = **{*z*_*ij*_}, λ=(λ_1_, …, λ_*m*_), τ = (τ_1_, …, τ_*n*_):


$$z_{ij}\{\begin{array}{c} r_{i}\cdot s_{j}\cdot e^{-1}\;if\;a_{ij}\geq 0\\ r_{i}^{-1}\cdot s_{j}^{-1}\cdot e^{-1}\;if\;a_{ij}<0 \end{array}$$

and the *e* is defined as $e^{\lambda_{i}}=r_{i}$ or simultaneously $e^{\tau_{j}}=s_{j}$. GRAS is proved to converge toward the minimum of error, stated with the following information loss problem:


$$x_{ij}\;=\;\arg\min\sum_{i}\sum_{j}\left|x_{ij}\right|\cdot ln\frac{x_{ij}}{a_{ij}}.$$

Number of iterations, and hence the time complexity, depends on the desired information loss value ϵ, which is typically provided by researcher. Alternatively, the fixed number of iterations can be specified, not relating to the desired information loss value ϵ. In our case, implementation of the GRAS was taken from the Matlab File Exchange website^5^ The algorithm was implemented by Temurshoev et al. (34).

We continued by steps 12–15 from original Jensen's numbering. These steps are devoted to further adjustments of the obtained regional I-O table. Furthermore, derivation of the inverse matrices is employed here:


$$L_{1}={\left(i-A^{\left(GRAS\right)}\right)}^{-1},$$

where the **A**^**(GRAS)**^ represents the technical coefficients matrix after balancing with the GRAS. The information loss function ensures that the **A**^**(GRAS)**^ is as much similar to **X**. The **A**^**(GRAS)**^ is enclosed with relation to households (endogenized) to form the Leontief type II inverse **B**^**(GRAS)**^. The first is to calculate the simple and type I multipliers, while the second to calculate the total/truncated and type II multipliers, as follows:


$$L_{2}={\left(i-B^{\left(GRAS\right)}\right)}^{-1}.$$

We have calculated five common different types of multipliers, for five different economic areas. These are outlined in the Table 3, following Miller and Blair (22) and as applied by Beko et al. (35) and Jagrič et al. (10). Several kinds of effects are captured by the multipliers. The simple multiplier captures direct and indirect, while the total type encloses also the induced effects. The direct effect is industry's initial economic impact on the economic ecosystem. The indirect effects come from transactions to other industries. Induced effects arise due to the enclosing the original IO table with additional industry sector, i.e., payments for labor services and consumer expenditure on goods, as explained by Miller and Blair (22). Next, the truncated multiplier considers the induced effects (such as total) but does not sum the additional sector on payments for labor services.

**Table 3:** Types of multipliers.

**Type of multiplier**	**Multiplier**	**Equation**
Output	Simple	$m\left(o\right)=m{\left(o\right)}_{j}=i^{\prime }\cdot L_{1}=\sum_{i=1}^{n}l_{ij}$
	Total	$\bar{m}\left(o\right)=\bar{m}{\left(o\right)}_{j}=i^{\prime }\cdot L_{2}=\sum_{i=1}^{n+1}{\bar{l}}_{ij}$
	Truncated	$\bar{m}\left[o\left(t\right)\right]=\bar{m}{\left[o\left(t\right)\right]}_{j}=i^{\prime }\cdot L_{2\left(11\right)}=\sum_{i=1}^{n}{\bar{l}}_{ij}$
Income	Simple	$m(h)=m{\left(h\right)}_{j}=h^{\prime }{}_{c}\cdot L_{1}={\sum^{\text{​}}}_{n}^{i=1}h_{i}\cdot l_{ij}$
	Total	$\bar{m}(h)=\bar{m}{\left(h\right)}_{j}={\bar{h}}^{\prime }{}_{c}\cdot [\begin{array}{c} L_{2(11)}\\ L_{2(21)} \end{array}]=\sum_{i=1}^{n+1}h_{i}\cdot {\bar{l}}_{ij}$
	Truncated	$\bar{m}[h(t)]=\bar{m}{\left[h(t)\right]}_{j}=h^{\prime }{}_{c}\cdot L_{2(11)}=\sum_{i=1}^{n}h_{i}\cdot {\bar{l}}_{ij}$
	Type I	$m{\left(h\right)}^{I}=m{\left(h\right)}_{j}^{I}=m(h)\cdot \;({\hat{h}}_{c}^{\prime })=\frac{m{\left(h\right)}_{j}}{h_{j}}$
	Type II	$m{\left(h\right)}^{II}=m{\left(h\right)}^{II}=L_{2(21)}\cdot \;{\left({\hat{h}}_{c}^{\prime }\right)}^{-1}=\frac{\bar{m}{\left(h\right)}_{j}}{h_{j}}$
Employment	Simple	$m(e)=m{\left(e\right)}_{j}=e^{\prime }{}_{c}\cdot L_{1}=\sum_{i=1}^{n}e_{i}\cdot l_{ij}$
	Total	$\bar{m}(e)=\bar{m}{\left(e\right)}_{j}={\bar{e}}^{\prime }{}_{c}\cdot [\begin{array}{c} L_{2(11)}\\ L_{2(21)} \end{array}]=\sum_{i=1}^{n+1}e_{i}\cdot {\bar{l}}_{ij}$
	Truncated	$\bar{m}[e(t)]=\bar{m}{\left[e(t)\right]}_{j}=e^{\prime }{}_{c}\cdot L_{2(11)}=\sum_{i=1}^{n}e_{i}\cdot {\bar{l}}_{ij}$
	Type I	$m{\left(e\right)}^{I}=m{\left(e\right)}_{j}^{I}=m(e)\cdot \;{\left({\hat{e}}_{c}^{\prime }\right)}^{-1}=\frac{m{\left(e\right)}_{j}}{e_{j}}$
	Type II	$m{\left(e\right)}^{II}=m{\left(e\right)}_{j}^{II}=L_{2(21)}\cdot \;{\left({\hat{e}}_{c}^{\prime }\right)}^{-1}=\frac{\bar{m}{\left(e\right)}_{j}}{e_{j}}$
Value-added	Simple	$m(d)=m{\left(d\right)}_{j}=d^{\prime }{}_{c}\cdot L_{1}=\sum_{i=1}^{n}d_{i}\cdot l_{ij}$
	Total	$\bar{m}(d)=\bar{m}{\left(d\right)}_{j}={\bar{d}}^{\prime }{}_{c}\cdot [\begin{array}{c} L_{2(11)}\\ L_{2(21)} \end{array}]=\sum_{i=1}^{n+1}d_{i}\cdot {\bar{l}}_{ij}$
	Truncated	$\bar{m}[d(t)]=\bar{m}{\left[d(t)\right]}_{j}=d^{\prime }{}_{c}\cdot L_{2(11)}=\sum_{i=1}^{n}d_{i}\cdot {\bar{l}}_{ij}$
	Type I	$m{\left(d\right)}^{I}=m{\left(d\right)}_{j}^{I}=m(d)\cdot \;{\left({\hat{d}}_{c}^{\prime }\right)}^{-1}=\frac{m{\left(d\right)}_{j}}{d_{j}}$
	Type II	$m{\left(d\right)}^{II}=m{\left(d\right)}_{j}^{II}=L_{2(21)}\cdot \;{\left({\hat{d}}_{c}^{\prime }\right)}^{-1}=\frac{\bar{m}{\left(d\right)}_{j}}{d_{j}}$
Import	Simple	$m(m)=m{\left(m\right)}_{j}=m^{\prime }{}_{c}\cdot L_{1}=\sum_{i=1}^{n}m_{i}\cdot l_{ij}$
	Total	$\bar{m}(m)=\bar{m}{\left(m\right)}_{j}={\bar{m}}^{\prime }{}_{c}\cdot [\begin{array}{c} L_{2(11)}\\ L_{2(21)} \end{array}]=\sum_{i=1}^{n+1}m_{i}\cdot {\bar{l}}_{ij}$
	Truncated	$\bar{m}[m(t)]=\bar{m}{\left[m(t)\right]}_{j}=m^{\prime }{}_{c}\cdot L_{2(11)}=\sum_{i=1}^{n}m_{i}\cdot {\bar{l}}_{ij}$
	Type I	$m{\left(m\right)}^{I}=m{\left(m\right)}_{j}^{I}=m(m)\cdot \;{\left(m_{c}^{\prime }\right)}^{-1}=\frac{m{\left(m\right)}_{j}}{m_{j}}$
	Type II	$m{\left(m\right)}^{II}=m{\left(m\right)}_{j}^{II}=L_{2(21)}\cdot \;{\left({\hat{m}}_{c}^{\prime }\right)}^{-1}=\frac{\bar{m}{\left(m\right)}_{j}}{m_{j}}$

Source: Beko et al. (35) and Jagrič et al. (21).

Type I and type II multipliers are relative measures, calculated as a ratio between simple multiplier and coefficient vector of compensation of employees (type I), or the total multiplier value and coefficient vector in case of type II.

For every type of the multiplier five different alternative estimates were calculated (on the level of significance α = 10%). Provided interval estimates can serve as an alternative measure of quality for estimated multipliers. Also, we use the results on interval estimates to enhance the interpretation of results. For each of the five alternative estimates, a (rank) per centile was calculated and, an average and standard deviation were derived upon. Assuming provided per centile estimates distribute normally, an interval with lower and upper bounds was calculated. The assumption of normal distribution of alternatives is though and empirically very difficult to prove. We must note that the taken approach is not founded on a specific statistical theory.

## Results

**Figure 1:**
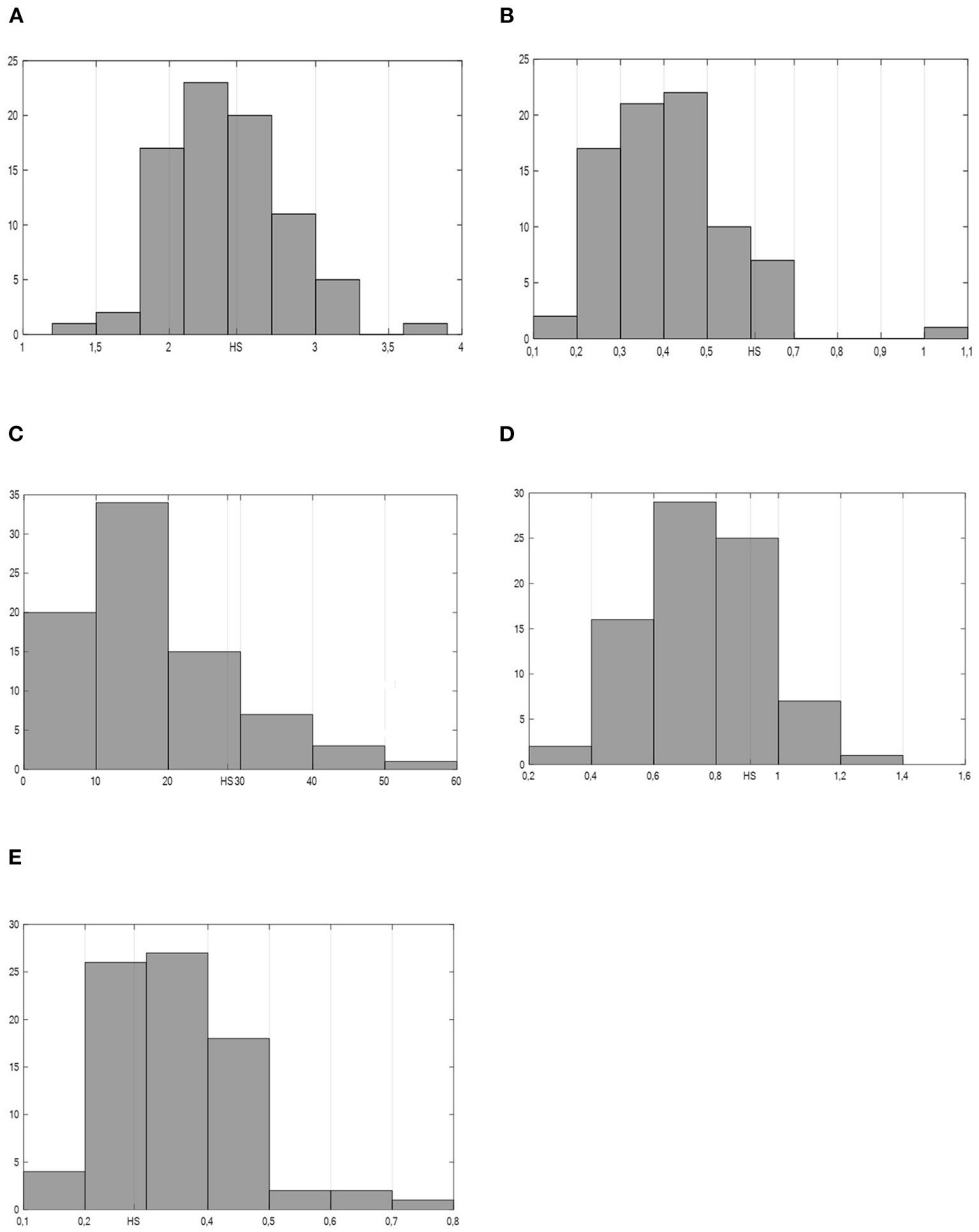
Distribution of total multipliers for all sectors—distribution (%) according to the multipliers' values. Position of the healthcare sector marked with HS. Source: Author's calculations. **(A)** Total output multiplier. **(B)** Total income multiplier. **(C)** Total employment multiplier. **(D)** Total value-added multiplier. **(E)** Total import multiplier.

Firstly, the comparison of the Welsh healthcare sector to other industries is given. The distribution of all total multipliers is shown in Figure 1. The abscissa represents magnitude of multipliers, while ordinate the frequency of multipliers. From the economic policy's perspective most favorable are high values for output, income, employment, and value-added multiplier. Contrary, for the import multiplier, the lowest values and highest rankings are most favorable, as this indicates the least leaking of economic impact through the imports. Our results indicate that in the comparison to our industries the position of the healthcare sector in the Welsh economy according to its impact on the local economy is above average in first four economic aspects (Figures 1A–D, while it is below average for the leaking through imports (Figure 1E).

### Output multipliers

We present the results on the output multipliers' estimations in Table 4: multipliers' values, ranks and percentiles. The rank reveals the comparison of the healthcare sector to other industries. The ranking of industries is made according to the multiplier‘s value, from the highest to the lowest one. Rank 1 would mean that an additional £1.00 spent for the industry's goods and services results in the most significant economic effects throughout the economy. The same industry will not necessarily have the same rank for all multipliers, depending on the economic ecosystem structure.

**Table 4:** Estimated values of output multipliers and rank of the healthcare industry in the ranking of all industries in the economic ecosystem.

				**Interval estimate (**α = **10%)**			
**Type**	**Value**	**Rank**	**Percentile**	**Average**	**St. dev**.	**L. bound**	**U. bound**
Simple	1.155937	73	92.4%	49.6%	43.8%	0.0%	100.0%
Total	2.466341	30	37.2%	20.7%	16.4%	0.0%	41.7%
Truncated	1.858659	47	59.0%	31.4%	28.0%	0.0%	67.3%

Interval estimates made under the assumption of normal distribution. Source: Author's calculations.

The simple multiplier is the lowest, however including induced effects (total multiplier) results in considerably higher estimated multipliers associated with the healthcare sector. Truncated multiplier's value is found between the simple and total multipliers.

Results show that simple multiplier for healthcare sector is in overall very low and ranks the healthcare sector at the 92nd percentile. The results on the total and truncated multiplier reveal another perspective. Oosterhaven et al. (36) argued true value of the given multiplier in real world lies between the values of simple and total multipliers. If final demand for services provided by the healthcare sector increases for £1.00, the overall output for whole economy increases app. by £2.47 when direct, indirect and induced effects are enclosed.

Additionally, an interval estimates are presented in Table 4. Results on the simple multiplier show that the interval estimate is very wide, while it gets narrower for the total multiplier. The truncated multiplier's interval is again wider.

### Income multipliers

Income multipliers show how the increased income of employees affects overall economy. Whether the employees receive higher compensation, they can spend more according to the marginal propensity to consume theory. This positively affects the industry sectors within an economy due to the increased demand. The results are given in Table 5. An increase of the final demand for the healthcare sector's services by £1.00 would cause the change of income in all sectors of the Welsh economy by ‘£0.50, considering only the direct and indirect effects, or for £0.61 when also including the induced effects.

**Table 5:** Estimated values of input multipliers and rank of the healthcare industry in the ranking of all industries in the economic ecosystem.

				**Interval estimate (**α = **10%)**			
**Type**	**Value**	**Rank**	**Percentile**	**Average**	**St. dev**.	**L. bound**	**U. BOuND**
Simple	0.494154	7	7.7%	12.1%	6.9%	3.2%	20.9%
Total	0.607682	7	7.7%	12.1%	6.9%	3.2%	20.9%
Truncated	0.607682	7	7.7%	12.1%	6.9%	3.2%	20.9%
Type I	1.067753	76	96.2%	65.0%	34.0%	21.4%	100.0%
Type II	1.313061	76	96.2%	65.0%	34.0%	21.4%	100.0%

Interval estimates made under the assumption of normal distribution. Source: Author's calculation.

The rank of the healthcare sector is seven in case of simple, total and truncated multipliers, meaning that income increments are within the top 10%, when all industries are compared. When considering interval estimation, it shows that healthcare sector ranks between the top 3 and 21%.

Increased compensation of employees in healthcare sector by £1.00 would cause an increase in the combined income across the economy of over £1.31. Again, the induced effects significantly contribute to the value. However, comparing relatively to other industry sectors, increase of compensation of employees only poorly contributes to increase of compensations in whole economy. The reason might be due to the high denominator, in other words, further increase of already high compensation in relative terms would not affect whole economy that much as an increase in large number of other sectors. However, the interval estimation is very wide therefore one must note the potential of overinterpretation when taking into account the wide interval estimate.

### Employment multipliers

Simple, total and truncated employment multipliers show how the increased demand in healthcare sector affects the number of employees in the whole economy. The results are presented in Table 6. Due to very low values, we express the change in final demand in millions of pounds. Thus, employment multipliers reveal how many new jobs are created in the whole economy, if the final demand for the healthcare sector's services increases by £1,000,000.

**Table 6:** Estimated values of employment multipliers and rank of the healthcare industry in the ranking of all industries in the economic ecosystem.

				**Interval estimate (**α = **10%)**			
**Type**	**Value**	**Rank**	**Percentile**	**Average**	**St. dev**.	**L. bound**	**U. bound**
Simple	23.00742	14	16.7%	12.6%	7.2%	3.4%	21.8%
Total	28.25989	14	16.7%	12.2%	7.0%	3.2%	21.1%
Truncated	28.25989	14	16.7%	12.2%	7.0%	3.2%	21.1%
Type I	1.105412	75	94.9%	82.5%	16.1%	61.9%	100.0%
Type II	1.357772	70	88.5%	79.8%	11.2%	65.4%	94.1%

Interval estimates made under the assumption of normal distribution. Source: Author's calculations.

Among all sectors in the economy, the healthcare sector is found at 14th rank or the 17th percentile, according to the estimated values of the simple, total and truncated employment multipliers. Otherwise, interval estimates forecast that the real population value should lie between 3 and 22%, for α = 10 %.

### Value-added multipliers

Simple, total and truncated value-added multipliers are all below one (see Table 7), which is in concordance with expectations based on the theoretical background. Induced effects have a moderate effect, as the rank of the total/truncated multiplier improves only slightly. Interval estimation shows that lower and upper bounds are found in the lowest two thirds of the multipliers in all cases, although there is increased uncertainty in the simple multiplier compared with the total/truncated multiplier estimates. It means that in the case of an increase in final demand for healthcare services of £1.00, there will be ~£0.91 of additional value-added in the whole Welsh economy, including households.

**Table 7:** Estimated values of value-added multipliers and rank of the healthcare industry in the ranking of all industries in the economic ecosystem.

				**Interval estimate (**α = **10%)**			
**Type**	**Value**	**Rank**	**Percentile**	**Average**	**St. dev**.	**L. bound**	**U. bound**
Simple	0.66264	28	34.7%	35.0%	15.8%	14.8%	55.3%
Total	0.913746	21	25.7%	25.2%	13.3%	8.2%	42.2%
Truncated	0.913746	21	25.7%	25.2%	13.3%	8.2%	42.2%
Type I	1.086816	74	93.6%	56.0%	39.2%	5.8%	100.0%
Type II	1.498663	62	78.3%	46.6%	34.8%	1.9%	91.2%

Interval estimates made under the assumption of normal distribution. Author's calculations.

The type I/II multipliers are calculated relative to initial effects. The results show that type I/II ranks are found in the last third in both cases, which means that increasing the value added in healthcare sector does not contribute much to the whole economy. However, one must note that the interval estimation is very wide, and as such point estimates should be interpreted with caution.

### Import multipliers

Finally, the results of estimated import multipliers are given in Table 8. Here, bear in mind, that lower values of multipliers are favorable reflecting the leakage effect as a part on the initial stimulus is lost for imports of inputs from other economic ecosystems. Further, note, that two different imports exist for geographic regions, i.e., international and interregional import, where statistical offices exhibit only the first among the two for a case of UK IO tables. Thus, interregional import needs to be estimated to some degree. Simple, total and truncated multipliers illustrates how the import for a whole Welsh economy changes, when the final demand for the healthcare sector's services increases by £1,00. Results for the simple multiplier denote that import increases to a minor degree only, ranking to the last third of all industry sectors. Induced effects add to the value of import multiplier, but do not affect much on the relative ranking to other sectors. Estimated standard deviation, and consequently interval bounds indicate that the results are reliable.

**Table 8:** Estimated values of import multipliers and rank of the healthcare industry in the ranking of all industries in the economic ecosystem.

				**Interval estimate (**α = **10%)**			
**Type**	**Value**	**Rank**	**Percentile**	**Average**	**St. dev**.	**L. bound**	**U. bound**
Simple	0.183835	58	73.1%	60.0%	12.2%	44.4%	75.6%
Total	0.288937	54	68.0%	53.4%	18.7%	29.3%	77.4%
Truncated	0.288937	54	68.0%	53.4%	18.7%	29.3%	77.4%
Type I	1.151574	70	88.5%	48.2%	36.1%	2.0%	94.5%
Type II	1.809952	47	59.0%	39.0%	19.1%	14.5%	63.4%

Interval estimates made under the assumption of normal distribution. Source: Author's calculations.

### Robustness check

To estimate whether the obtained results are robust we were benchmarking them. As a benchmark value we used a forecasted value derived from econometric models. These econometric models, developed on results for 19 European countries on I-O models for 2010, are found in an international study (21).

**Figure 2:**
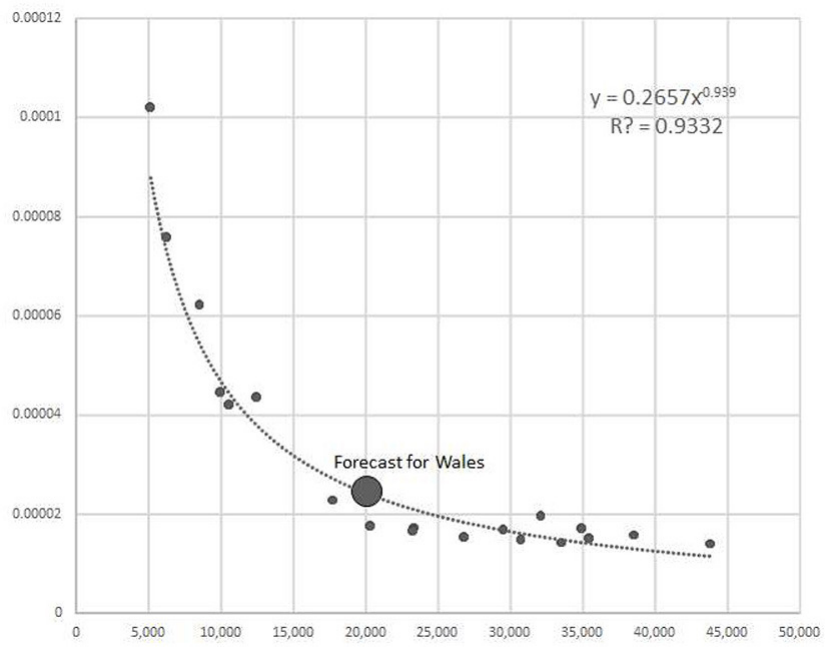
Forecast of simple employment multiplier for Wales based on the econometric model. Source:Author's calculations and (10).

To consider the employment multiplier, one can note that the model reveals a remarkable, strong, non-linear negative relationship between GDP per capita and the employment multiplier based on the international data (see Figure 2). In this study, we use the model from the previous study and forecast the Welsh multipliers' value. Next, we compare the forecasted value to the here obtained results. When using the model to derive an expected value, we find that the latter is remarkably close to the real data results from this study. Namely, the benchmarking model forecasted the value of the simple employment multiplier to be 24,67, while in this study empiricaly estimated value based on I-O table resulted in 23,01, both per an impulse of additional demand of £1.000.000. Such convergence between the two speaks for reasonable results of the present study.

## Discussion

In the literature, there are several measures discussed regarding the healthcare sectors' transformation in various countries (4, 6, 17, 19, 37–39). As in this study we explore the economic impact, we further discuss how the economic impact on the local economic ecosystem could be strengthen further. Applying some of the measures of the health policy from the above literature could namely support the Welsh healthcare sectors' economic interaction with the local economic ecosystem and thereby support the Welsh economic development in a post-COVID and post-Brexit period, such as:

- Strengthening access to healthcare in Welsh local environments through expanding services, e.g., including secondary outpatient services, equal distribution of services by areas, improving availability and access, broadening preventive healthcare programs.
- Promoting a healthy and productive workforce to support regional economic development. Measures to support them include social measures to protect job and income security, promoting positive health-related behaviors.
- Supporting a viable and robust local healthcare sector by preserving local healthcare financing sources being spent within the Welsh economic ecosystem and attracting additional demand from aboad bringing income into the industry and closing supply-demand gaps.
- Promoting e-health by implementing new support and reimbursement mechanisms for easing patient access and promoting digital health literacy, build data policies to allow artificial-intelligence to support diagnosis and treatment decisions, and improving resource and capacities usage.

The obtained multipliers estimate an effect of additional final demand for the healthcare services provided by the Welsh healthcare sector as a whole. Evidence in other countries shows that the effect differs considerably by a single provider, and by region or municipality (10). The multipliers' values differ due to differences in its interconnection to the surrounding economic ecosystem. Therefore, it would be of benefit to study individual parts of the healthcare sector, such as an individual hospital. When doing so, a combination of input-output analysis and analysis of primary survey data could be used.

## Conclusions

As with any study, the results must be interpreted properly considering data and methodological characteristics and in the context of assumptions and limitations. Firstly, the here presented study is an economic study, therefore only economic effects are reflected. Therefore, no other kind of effects are considered, including changes in direct health outcomes that may result from changes in final demand of the healthcare sector's services. In interpreting the results, this should be taken into account, therefore discussing the perspective of the economic ecosystem only.

Further, there are assumption and limitations arising from the methodological regard in the economic research context. One should bear in mind that the study derives from a static model, reflecting the given moment in time. Additionally, there are assumed constant returns to scale, no supply constraints, fixed input structure, industry technology assumption and constant make matrix. Several further issues may affect results, such as transportation issues, tax policy, price changes and forward linkages, and significant structural changes. When the results are used, the characteristics of the here analyzed Welsh health sector in regard to the named assumptions have to be taken into account.

Another very important perspective of the results' interpretation comes from the fact that the study, contrary to many other economic studies does not express nor does it measure the effectiveness of health expenditures (e.g., intra-institutional effectiveness, organizational governance). Further, the results do not reflect the return and are therefore not a measure of return-on-investment.

The data used in the study is from the year 2017. The results are point-in-time estimated and therefore hold for the year explored and reflect the structure of the Welsh economy in the year 2017, respectively. Several factors might have caused the position of the healthcare sector in the Welsh economic ecosystem to be different at present, to name a few, business cycle, COVID-19 and Brexit. Nevertheless, the result can be interesting in the international context; namely, Wales is an example of a small high-income country. As we discussed in the study background, the literature gives reasons to believe that, although individual cases of countries differ, some common patterns are indicated.

The presented study aims to quantify the economic “footprint” of the Welsh healthcare sector to the wider economy in Wales. It expresses the economic impact of a change in final demand. The local healthcare sector reacts to this change either by providing additional services or its reduction. The economic impact is seen by the knock-on effect on the whole economic ecosystem. We monitor the impact by measuring the effects in the following dimensions: the economy‘s output, value-added, income, employment, and import. The study suggests several policy implications based on the estimated results.

The healthcare sector in Wales is vital in maintaining population health and wellbeing, but also is a significant sector of the economy, capable of stimulating economic output through investment and job creation. Using multipliers to show the contribution of a sector (in this case, the healthcare sector) to a national/regional economy can help decision/policy-making and investment prioritization within limited budgets. In this case, comparison to other industries (sectors) is interesting since often an alternative choice of projects or programmes can be financed due to limited public resources. Sectoral ranking compares the values of healthcare sectors' multipliers to other industries. Higher ranks suggest that an additional final demand for the industry's goods and services results in the most significant economic effects when measured as output, income, employment, or value-added change throughout the economy. Policy/investment decisions can also have an impact on the multipliers' values to change, such as by intensifying the interconnectedness of the healthcare providers to the local economy. Healthcare providers thus strengthen their role as anchoring institutions to local economies and communities.

The Welsh healthcare sector is consistently amongst the most influential sectors when across the entire range of economic measures is explored. Service sectors make up the top ten industries of the Welsh economy according to the ranking by total output, total income, total value-added or total employment multiplier. Rankings by individual multiplier differ. All total multipliers (output, income, value-added, employment) rank the healthcare sector above the Welsh economy‘s average. The comparison from simple to total multipliers indicate, that in the case of the Welsh healthcare sector, an important part of the economic effects of the healthcare sector come as induced effects, namely, from household spending from income.

Regarding the leaking of economic effects through imports we found that none of the import multipliers (simple, total and truncated) is greater than the average in the economy. Greater demand in other sectors will, to a more significant extent, cause imports to rise compared to the effect of the same change in demand for services of the health sector. From the economic perspective, we argue that there are prevailing positive effects on the Welsh economy if the final demand for healthcare sectors' products and services rises, especially when compared with the impact of the same changes in other sectors.

These results challenge misperceptions of the healthcare sector as an economic drain, rather than a powerful stabilizer and investment multiplier. As Wales moves into the recovery period from the Coronavirus pandemic and faces challenges from Brexit and climate change, there is a significant opportunity to use investment in the healthcare sectors' services as an engine for a sustainable and equitable economic recovery toward creating an economy of wellbeing for all.

## Data availability statement

The original contributions presented in the study are included in the article/supplementary materials, further inquiries can be directed to the corresponding author/s.

## Author contributions

TJ and CB: conceptualization. TJ: methodology, validation, formal analysis, visualization, and supervision. TJ and DF: data gathering and software. DF and VJ: writing—original draft preparation. CB, OD, KA, MD, and VJ: writing—review and editing. All authors contributed to the article and approved the submitted version.

## Funding

This study was funded by Public Health Wales.

## Conflict of interest

The authors declare that the research was conducted in the absence of any commercial or financial relationships that could be construed as a potential conflict of interest.

## Publisher's note

All claims expressed in this article are solely those of the authors and do not necessarily represent those of their affiliated organizations, or those of the publisher, the editors and the reviewers. Any product that may be evaluated in this article, or claim that may be made by its manufacturer, is not guaranteed or endorsed by the publisher.

## Footnotes

^1^For more see https://www.futuregenerations.wales/about-us/future-generations-act/.

^2^For more see https://gov.wales/more-equal-wales-socio-economic-duty#:~:text=The%20Socio%2Deconomic%20Duty%20came,do%20things%20differently%20in%20Wales.

^3^For more see https://gov.wales/programme-government.

^4^For more see https://gov.wales/healthier-wales-long-term-plan-health-and-social-care.

^5^https://www.mathworks.com/matlabcentral/fileexchange/43231-generalized-ras-matrix-balancing-updating-biproportional-method.
